# Calendar

**DOI:** 10.1080/15604280212529

**Published:** 2002


					INTERNATIONAL JOURNAL OF EXPERIMENTAL DIABETES RESEARCH
75
February 08, 2002
Diabetes and the Heart, The
Burns Lecture and The
Flemming Lecture
Glasgow, Scotland, UK
Contact: Royal College of
Physicians and Surgeons of
Glasgow, 232-242 St Vincent
Street, Glasgow G2 5RJ
Gayle McKeever
Tel: 01-412-216-072
Fax: 01-412-211-804
e-mail:
gmckeever@rcpsglasg.ac.uk
February 16-18, 2002
Diabetes and the Eye 2002
Rancho Mirage, CA, USA
Contact: Diane Evans
Tel: +1-760-773-4514
Fax: +1-760-773-4550
e-mail:
registration@annenberg.net
March 02, 2002
Diabetes Symposium
Springfield, IL, USA
Contact: School of Continuing
Education
e=mail: bshelow@siumed.edu
March 7-10, 2002
8th International Conference:
Diabetes & Obesity in conjunc-
tion with the University of the
West Indies: Diabetes Outreach
Procejct (UDOP), The Pan
American Health Organisation
(PAHO), The American
Diabetes Association and The
Caribbean Food and Nutrition
Institute
Ocho Rios, Jamaica
Contact: Ms. Thornia Smith
Tel: +1-876-977-1749
Fax: +1-876-977-5233
e-mail:
udop@uwimona.edu.jm
March 20-22, 2002
5 Congresso Portugues de
Diabetes
Vilamoura-Mariontel,
Portugal
Contact: K.I.T., a/c Sociedade
Portuguesa de Diabetologia,
Praca Marques de Pombal, 16A
- 5 Piso
Tel: +351-213-504064
Fax: + 351-213-504024
e-mail: llouro@kit.de
March 26-29, 2002
6th Pan Arab Conference on
Diabetes: PACD 6
Cairo, Egypt
Contact: Dr. Mahmoud Ashraf
Mohamed
Tel: 20-122-131-868
Fax: 2-022-723-693
e-mail:
mahmoud@arab-diabetes.com
June 15-18, 2002
62nd Annual Scientific Sessions
of the American Diabetes
Association
San Francisco, CA, USA
Contact: ADA's Meetings
Department
Tel: +1-703-549-1500 ext.3553
e-mail: meetings@diabetes.org
September 1-5, 2002
Annual Conference of the
European Association for the
Study of Diabetes
Budapest, Hungary
Contact: Meeting Organizer
Tel: 49-0-6050-97220
Fax: 49-0-6050-972-210
e-mail: PS@profi-sales.com
October 2-6, 2002
6th International Congress of
Immunology of Diabetes
Society and ADA Research
Symposium
Copper Mountain, CO, USA
Contact: ADA's Meetings
Department
Tel: +1-703-549-1500
ext. 2134
e-mail: meetings@diabetes.org
October 24-27, 2002
EASD Islet Study Group
N. Falmouth, MA, USA
Contact: ADA's Meetings
Department
Tel: +1-703-549-1500 ext.
2134
e-mail: meetings@diabetes.org
C A L E N D A R
Please send
conference and
symposium
listings to:
628 N. 2nd Street
Philadelphia, PA,
19123, USA

				

